# Clinical and pathological analysis of renal biopsies of elderly patients in Northeast China: a single-center study

**DOI:** 10.1080/0886022X.2021.1923527

**Published:** 2021-05-10

**Authors:** Ping Nie, Yan Lou, Yali Wang, Xue Bai, Li Zhang, Shan Jiang, Bing Li, Ping Luo

**Affiliations:** The Department of Nephropathy, The Second Hospital of Jilin University, Changchun, China

**Keywords:** renal biopsy, elderly, primary glomerulonephritis, pathology, prognosis

## Abstract

**Purpose:**

To identify the clinical characteristics, histopathological features, and prognosis of kidney disease in a large cohort of elderly patients from Northeast China.

**Methods:**

We retrospectively analyzed the renal disease spectrum in 7,122 patients who underwent renal biopsies at the Second Hospital of Jilin University from 2006 to 2020. Patients were grouped according to age: below 60 years (non-elderly group, *n* = 5923) and at least 60 years (elderly group, *n* = 1199). The clinical and pathological characteristics of renal biopsy patients in the groups were analyzed using the *t*-test and chi-square test.

**Results:**

Compared with the non-elderly group, the elderly group had significantly fewer patients with primary glomerulonephritis, but more patients with tubulointerstitial disorders (*p* < .05). The incidence of IgA nephropathy, mesangial proliferative glomerulonephritis, and lupus nephritis was significantly lower in elderly patients than in non-elderly patients. The incidence of membranous nephropathy, membranoproliferative glomerulonephritis, diabetic nephropathy, hypertensive nephropathy, systemic vasculitis-associated renal damage, and amyloid nephropathy was significantly higher in elderly patients than in non-elderly patients (*p* < .05). The incidence of perinephric hematoma (≥4 cm^2^) in elderly patients with renal biopsy was lower than that in non-elderly patients. We noted that 79.9% of primary glomerulonephritis patients who received immunosuppressive therapy showed a remission rate of 83.5%.

**Conclusion:**

The spectrum of kidney disease in the elderly is different from that in the younger population.

## Introduction

The prevalence of chronic kidney disease (CKD) has increased rapidly, and the incidence rate is highest in elderly patients (up to 38%) [1]. The elderly are more prone to develop kidney diseases due to the presence of co-morbidities such as diabetes and hypertension [2–4]; further, the treatment plan for them varies from that for young people.

Renal histopathological analysis is the gold standard for the diagnosis of renal diseases. Percutaneous renal biopsy, which was performed first by Iversen and Brun in 1951, is used by clinicians worldwide [5]. The complication rate after renal biopsy is low, and older age alone is not a contraindication to biopsy. In the absence of significant hypertension or bleeding tendency, the incidence of complications due to renal biopsy is observed to be 7–8%, regardless of the patient's age [1,6]. The condition of elderly patients with kidney disease is complicated. Renal biopsy, which can identify the pathological changes in patients, is the key to appropriate management of elderly patients with kidney disease [7]. Therefore, it is necessary to study the clinical and pathological characteristics of nephropathy in elderly patients to help determine an appropriate treatment.

Studies on the pathological types of renal diseases in elderly patients in Northeast China based on biopsy data are very limited. There are few studies that have been performed on large sample sizes. However, due to the population outflow, the situation of the aging population in Northeast China is rather grave [8]. Analysis of the pathological types of renal diseases in elderly patients can play a guiding role in their treatment. Therefore, it is of great significance to study the renal biopsy data of elderly patients in Northeast China. Since our center has performed the largest number of renal biopsies in Jilin Province, China, we compared the clinical and pathological characteristics of 1199 elderly patients and 5923 non-elderly patients who underwent renal biopsies in our hospital from 1 January 2006, to September 30, 2020. We also analyzed the complications, treatment protocols, and the prognosis in elderly patients between May 2015 and April 2017.

## Materials and methods

### Patients

This retrospective study was performed on all the patients who underwent renal biopsy at the Second Hospital of Jilin University from January 2006 to September 2020. During this period, a total of 7,122 renal biopsies were performed; of these, 1,199 were in patients aged over 60 years and 5,923 were in patients aged less than 60 years. Each participant signed an informed consent form before the biopsy. This study was approved by the ethics committee of our hospital (2020100). Written informed consent was obtained from the patients for their participation and the use of their data for analysis.

All the patients in this study underwent color doppler ultrasound-guided percutaneous renal biopsy to obtain 2–3 renal tissue samples. In most patients, the biopsy was performed using a 16-gauge needle; however, in patients with high serum creatinine levels and hypertension, an 18-gauge needle was used to reduce the risk of bleeding. Biopsy was not performed in patients with severe thrombocytopenia and coagulopathy.

### Clinical manifestations

The clinical manifestations observed were classified as glomerulonephritis syndrome (GN), nephrotic syndrome (NS), acute kidney injury (AKI), and chronic kidney disease (CKD). GN is characterized by hematuria, urinary red blood cell casts, proteinuria (<3.5 g.day^−1^), and hypertension. NS is characterized by proteinuria (≥3.5 g.day^−1^), plasma albumin level <30 g.L^−1^ with edema, and hyperlipidemia. AKI is defined as an increase in serum creatinine by 26.5 μmol.L^−1^ or an increase in serum creatinine by 50% during hospital stay [9]. CKD is defined as a decrease in glomerular filtration rate (GFR) (<60 mL·min^−1^·1.73 m^−2^) for more than three months. According to the Kidney Disease Improving Global Outcomes (KDIGO), treatment effects were classified into complete remission (CR), partial remission (PR), and no response (NR). CR was defined as a urinary protein excretion of ≤0.3 g.day^-1^, accompanied by normal serum concentrations of albumin and SCr. PR was defined as the urinary protein excretion between 0.3 g.day^-1^ and 3.5 g.day^-1^, and improvement or normalization of serum albumin concentration, a stable level of SCr. NR was defined as no improvement in urine protein excretion and serum albumin levels.

### Pathological classification

All paraffin-cut sections were stained with hematoxylin and eosin, periodic acid-Schiff, periodic acid-Schiff-methenamine, Masson’s trichrome solution, and Congo red for examination under a light microscope. Frozen tissues were stained with IgA, IgM, IgG, C3, C4, C1q, fibrinogen, kappa, and lambda light chains for immunofluorescence analysis. Further, electron microscopic examination was performed.

Renal histopathological diagnosis was divided into four main categories: primary glomerulonephritis (PGN), secondary glomerular disease (SGN), tubulointerstitial nephropathy (TIN), and hereditary nephropathy (HN) according to the 1995 World Health Organization histological classification of glomerular diseases [10]. PGN included IgA nephropathy (IgAN), mesangial proliferative glomerulonephritis (MsPGN), membranous nephropathy (MN), focal segmental glomerulosclerosis (FSGS), minimal change disease (MCD), glomerular minor lesion (GML), crescentic glomerulonephritis (CreGN), membranoproliferative glomerulonephritis (MPGN), endocapillary proliferative glomerulonephritis (EnPGN), proliferative sclerosis and sclerosing glomerulonephritis. SGN included lupus nephritis (LN), Henoch-Schönlein purpura nephritis (HSPN), hepatitis B virus-associated nephritis (HBV-GN), diabetic nephropathy (DN), systemic vasculitis-associated renal damage (SVARD), malignant/hypertensive nephropathy (M/HTN), dysproteinemic kidney diseases (amyloid nephropathy [AN] and light chain deposition nephropathy [LCDD]), obesity-associated glomerulopathy (OAG), and others (C3 glomerulopathy, hepatitis C virus-associated nephritis, etc.). TIN included acute interstitial nephritis (AIN), acute tubular necrosis (ATN), chronic interstitial nephritis (CIN), and subacute tubulointerstitial lesions. HN included thin basement membrane nephropathy, Alport syndrome, Fabry disease, and collagen III glomerulopathy. For the cases with two or more pathological changes, we carefully analyzed the pathological and clinical manifestations and selected the most important pathological diagnosis for statistical analysis.

### Statistical analysis

We used SPSS version 25.0 (Chicago, IL, USA) for data analysis and GraphPad Prism 7 (GraphPad, La Jolla, USA) for graphical analysis. Continuous variables were presented as mean ± standard deviation. *T*-tests were used to compare the differences between the two groups. Comparisons among the three age groups were performed using one-way ANOVA. Categorical variables were presented as percentages and were compared using the chi-square test. A *p*-value of less than .05 was considered significant.

## Results

### Demographic data of elderly patients with renal biopsy

**Figure 1.**
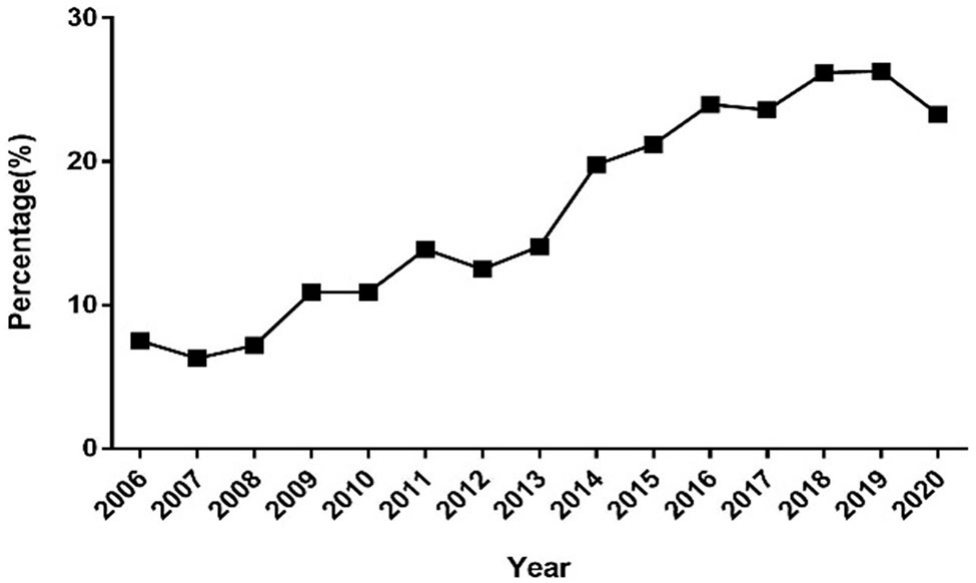
Annual percentage of elderly patients (≥60 years old) who underwent renal biopsy from 2006 to 2020.

Among a total of 7,122 renal biopsy specimens, 1,199 (16.8%) specimens were obtained from elderly patients (aged ≥60 years) in this study. The proportion of elderly patients who underwent renal biopsy significantly increased over time, exceeding 20% in 2015 and peaking at 26.3% in 2019 (Figure 1). Concurrently, 5,923 renal biopsy specimens were obtained from non-elderly patients (aged < 60 years).

The elderly patients comprised 704 men and 495 women (male: female ratio, 1.42:1) with a mean age of 66.4 ± 5.3 years (range, 60–84 years). Non-elderly patients consisted of 3,113 men and 2,810 women (male: female ratio, 1.11:1) with a mean age of 38.2 ± 12.8 years. The proportion of male patients was significantly higher in elderly patients (*p <* .001).

### Clinical manifestations of kidney disease in different age groups

**Figure 2.**
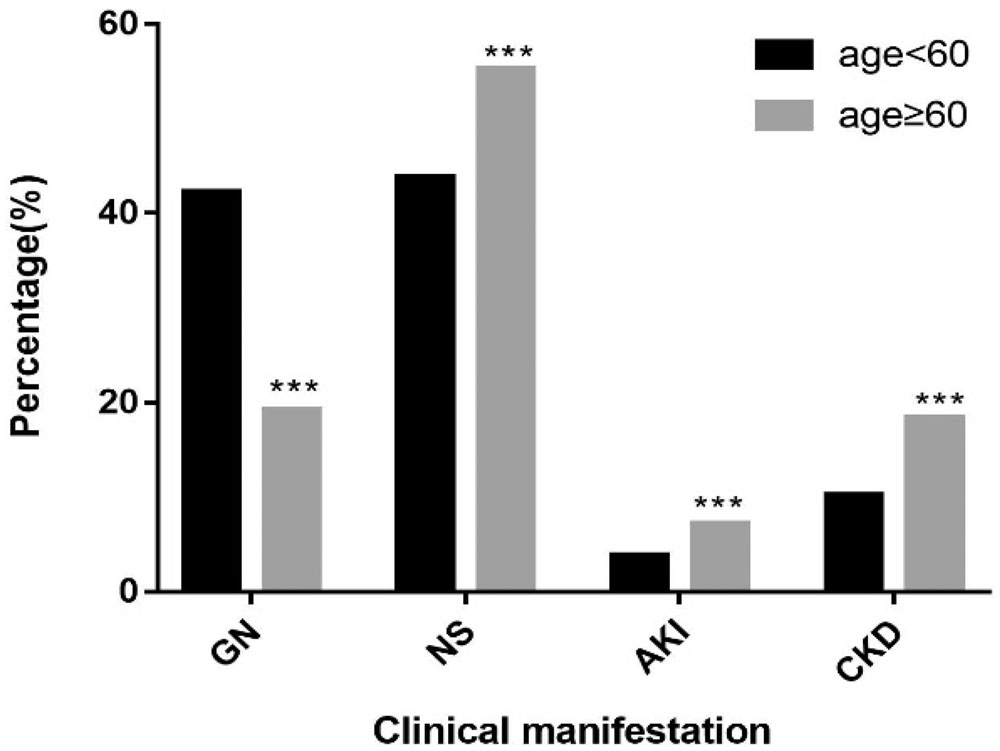
Clinical manifestations of renal biopsy in different age groups. *p < .05, **p < .01, ***p < .001 vs non elderly group by Chi-square test. *Abbreviations: GN*, glomerulonephritis; *NS*, nephrotic syndrome; AKI: acute kidney injury; CKD: chronic kidney disease.

Among the elderly patients who underwent renal biopsy, NS (55.2%) was the most common manifestation, followed by GN (19.3%), CKD (18.3%), and AKI (7.2%). The clinical manifestations of non-elderly patients were NS (43.8%), GN (42.2%), CKD (10.2%), and AKI (3.8%). Compared with that in non-elderly patients, the incidence of GN in elderly patients were significantly lower and that of NS, CKD, and AKI were significantly higher (*p <* .001, respectively; Figure 2).

### Complications of renal biopsy in different age groups

We analyzed the postoperative complications of 901 patients who underwent renal biopsy from May 2015 to April 2017 (Table 1). Serious complications (defined as death or as requiring a blood transfusion, surgery, arteriography, or nephrectomy) were absent in both groups. The red blood cells in urine of 46 elderly patients (22.3%) increased more than three times after renal biopsy, however, only one elderly patient had gross hematuria. Perirenal hematoma (≥4 cm^2^) was observed in 23 elderly patients (11.2%) and in 189 non-elderly patients (27.2%), showing a significant difference (*p* < .001). The other complications included perirenal hematoma (<4 cm^2^), urinary retention, and intolerant lumbago or abdominal distention seen in 39 (18.9%), 11 (5.3%), and 8 (3.9%) elderly patients, respectively.

**Table 1.** Minor complications of renal biopsy in different age groups between May 2015 and April 2017.

complications	≥60 years old	<60 years old	*p*-value
	*N* = 206	*N* = 695	
Microscopic hematuria	46 (22.3)	138 (19.9)	.439
Gross hematuria	1 (0.5)	2 (0.3)	1.000
Perirenal hematoma (<4cm^2^)	39 (18.9)	112 (16.1)	.342
Perirenal hematoma (≥4cm^2^)	23 (11.2)	189 (27.2)	<.001***
Pain of waist or abdomen	8 (3.9)	48 (6.9)	.114
Urinary retention	11 (5.3)	47 (6.8)	.465

Complications of renal biopsy in different age groups between May 2015 and April 2017. ***p < .001 by the chi-square test.

### Pathological types of kidney disease in different age groups

**Figure 3.**
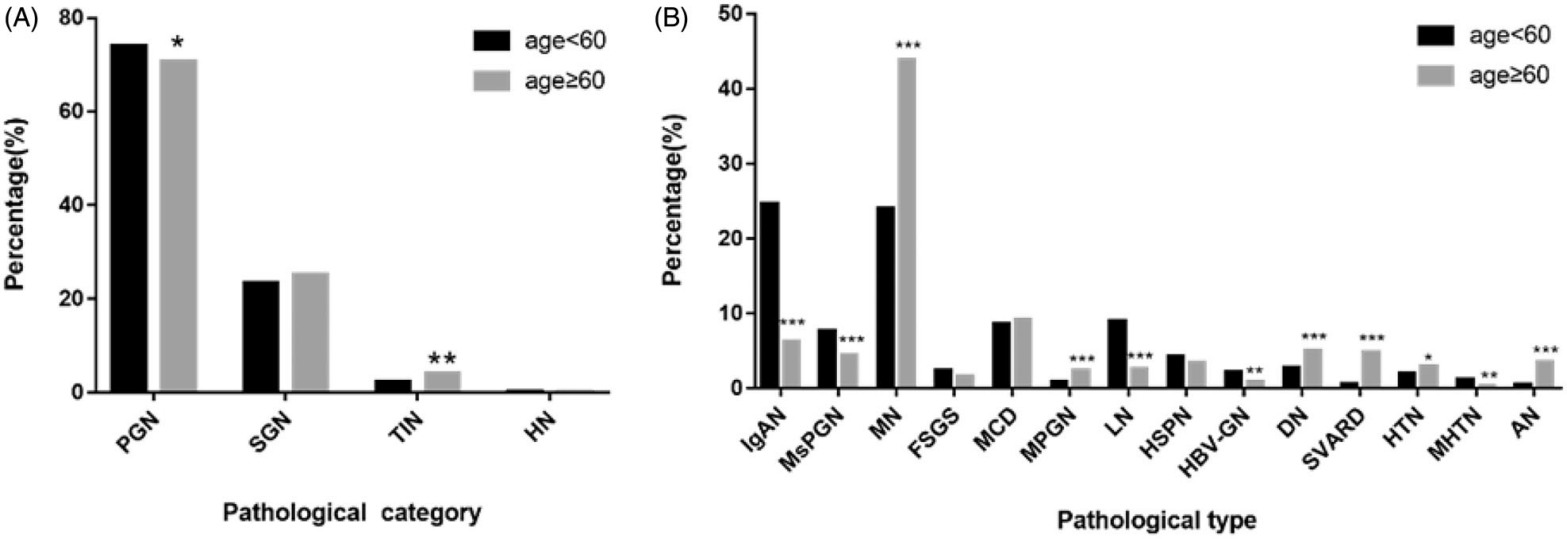
(A) Distribution of pathological categories in different age groups. (B) Distribution of common pathological types in different age groups. *p < .05, **p < .01, ***p < .001 vs non-elderly group by Chi-square test.

The most common histopathology observed in the biopsy specimens of both groups was PGN; however, it was significantly lower in the elderly (70.8%) than that in the non-elderly patients (74%) (*p =* .023). This was followed by SGN, present in 25.2% of the elderly and 23.4% of the non-elderly patients, however, the difference was not statistically significant. TIN was significantly higher in the elderly (3.9%) than in the non-elderly patients (2.4%) (*p =* .002). The incidence rate of HN in both groups was low (Table 2 and Figure 3(A)).

**Table 2.** Pathological types of kidney diseases in different age groups.

Pathology types	≥60 years-old	<60 years-old	*p* value
	*N* (%)	*N* (%)	
***PGN***	849 (70.8)	4382 (74.0)	.023*
IgAN	75 (6.3)	1,464 (24.7)	<.001***
MsPGN	52 (4.3)	456 (7.7)	<.001***
MN	526 (43.9)	1,419 (24.0)	<.001***
FSGS	19 (1.6)	143 (2.4)	.079
GML	7 (0.6)	130 (2.2)	<.001***
MCD	110 (9.2)	508 (8.6)	.503
CreGN	14 (1.2)	43 (0.7)	.118
MPGN	28 (2.3)	54 (0.9)	<.001***
EnPGN	4 (0.3)	27 (0.5)	.558
sclerosis	14 (1.2)	138 (2.3)	.011*
***SGN***	302 (25.2)	1384 (23.4)	.176
LN	31 (2.6)	535 (9.0)	<.001***
HSPN	41 (3.4)	254 (4.3)	.169
HBV-GN	11 (0.9)	127 (2.1)	.005**
DN	61 (5.1)	162 (2.7)	<.001***
SVARD	58 (4.8)	38 (0.6)	<.001***
HTN	35 (2.9)	113 (1.9)	.025*
MHTN	4 (0.3)	71 (1.2)	.007**
AN	42 (3.5)	31 (0.5)	<.001***
LCDD	4 (0.3)	13 (0.2)	.679
OAG	0 (0)	13 (0.2)	.144
IN	5 (0.4)	7 (0.1)	.056
ING	6 (0.5)	6 (0.1)	.007**
Others	4 (0.3)	14 (0.2)	.767
***TIN***	47 (3.9)	140 (2.4)	.002**
***HN***	1 (0.1)	17 (0.3)	.334

PGN: primary glomerulonephritis; IgAN: IgA nephropathy; MsPGN: mesangial proliferative glomerulonephrilis; MN: membranous nephropathy; FSGS: focal segmental glomerulosclerosis; GML: glomerular minor lesion; MCD: minimal change disease; CreGN: crescentic glomerulonephritis; MPGN: membrano-proliferative glomerulonephritis; EnPGN: endocapillary proliferative glomerulonephritis; sclerosis, proliferative sclerosis and sclerosing glomerulonephritis. SGN: secondary glomerulonephritis; LN: lupus nephritis; HSPN: Henoch-Schönlein purpura nephritis; HBV-GN: hepatitis B virus associated nephritis; DN: diabetic nephropathy; SVARD: systemic vasculitis-associated renal damage; (M)HTN*:* (malignant) hypertensive nephropathy; AN: amyloidosis nephropathy; LCDD: light chain deposition nephropathy; OAG: obesity-associated glomerulopathy; IN: Ischemic nephropathy; ING: idiopathic nodular glomerulosclerosis; Others: other secondary glomerulonephritis; TIN: tubular-interstitial nephropathy; HN: hereditary nephropathy.

*p < .05, **p < .01, ***p < .001 by the chi-square test.

The distributions of the common pathological types of kidney disease were shown in Figure 3(B). In the elderly patients, PGN was commonly seen as MN (43.9%), MCD (9.2%), and IgAN (6.3%). In addition, it was observed that the proportions of elderly patients with IgAN, MsPGN, and GML were significantly lower than those of non-elderly patients (*p <* .001 each). In comparison, the incidence of MN and MPGN was significantly higher among elderly patients than among non-elderly patients (*p <* .001 each).

In the elderly patients, SGN commonly presented as DN (5.1%), SVARD (4.8%), and AN (3.5%). The incidence rates of DN, SVARD, HTN, AN, and ING in the elderly patients were significantly higher than those in the non-elderly patients (*p* < .001, *p* < .001, *p* = .025, *p* < .001, and *p* = .007, respectively), and the incidence rates of LN, MHTN, and HBV-GN in the elderly patients were lower than those in the non-elderly patients (*p* < .001, *p* = .007, *p* = .005, respectively) (Table 2 and Figure 3(B)).

In the elderly patients, TIN presented as 20 cases of subacute TIN, 14 cases of ATN, 10 cases of AIN, and 3 cases of CIN. The incidence rates of ATN and subacute TIN in the elderly patients were higher than those in the non-elderly group (*p* = .010 and *p* = .004, respectively).

### Clinical indicators and pathological types of elderly patients who underwent renal biopsy

Following laboratory investigations, the observed mean values of blood albumin and proteinuria, in the elderly patients, were 27.5 ± 9.1 g.L^−1^ and 5.2 ± 3.5 g.day^−1^, respectively. Renal function was normal in 74.5% of the elderly patients.

**Figure 4.**
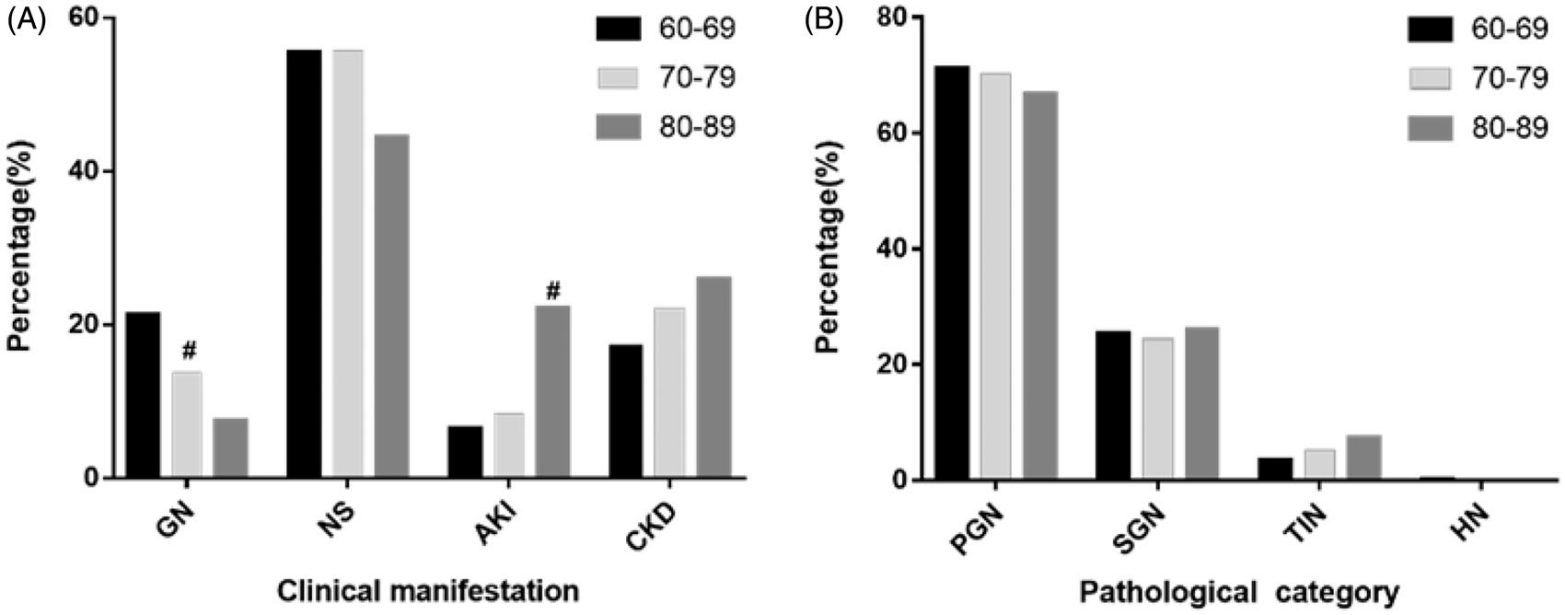
(A) Distribution of clinical categories in different age groups of elderly patients. (B) Distribution of pathological categories in different age groups of elderly patients. #*p* < .017 vs 60–69 years group by Chi-square test. There were significant differences in GN (*p* = .007) and AKI (*p* = .022) among three age groups. We further compared every two groups, and *p* < .017 was considered statistically significant. Compared with that in the 60–69 age group, the incidence of GN in the 70–79 age group decreased significantly (*p* = .008) and the incidence of AKI in the 80–89 age group increased significantly (*p* = .008).

The elderly patients were further categorized into three groups: 60–69 years old (910 patients, 75.9%), 70–79 years old (262 patients, 21.9%), and 80–89 years old (27 patients, 2.3%). No significant difference was observed in blood albumin and urinary protein levels among the three groups. With an increase in age, the incidence of GN significantly decreased, and the incidence of AKI significantly increased (*p* = .007 and *p* = .005, respectively; Figure 4(A)). We further compared every two groups among them. Compared with that in the 60–69 age group, the proportion of patients with GN in the 70–79 age group was significantly decreased (*p* = .008) and that of AKI in the 80–89 age group was significantly increased (*p* = .008). Among the patients aged 80–89 years, 6 (22.2%) were diagnosed with AKI; of these patients, 2 had SVARD, 1 had ATN, 1 had CreGN, and 2 had MCD with severe edema.

No significant differences were observed in relation to the pathological types found among the three groups, Among the 27 patients over 80 years old, 18 patients had PGN (66.7%), 7 had SGN (25.9%), and 2 had TIN (7.4%) (Figure 4(B)).

### Data for treatment and prognosis

As shown in Table 3, between May 2015 and April 2017, 79.9% of patients with PGN received immunosuppressive therapy and showed a remission rate of 83.5%. The most common forms of PGN, MN and MCD, were diagnosed in 103 and 23 patients, respectively. In 49 patients with MN, a combination of glucocorticoids and cyclophosphamide (CTX) was administered; among these patients, 18 achieved complete response (CR) and 19 achieved partial response (PR), resulting in a remission rate of 82.2%. However, 8 patients with MN showed no response (NR) to treatment and three patients developed infection. Calcineurin inhibitors (CNIS) mainly comprised ciclosporin A (CSA) and tacrolimus (TAC). A combination of glucocorticoids and TAC was administered to 12 patients, and a combination of glucocorticoids and CSA was administered to 10 patients; of these, 6 and 11 patients achieved CR and PR, respectively, resulting in a remission rate of 85.0%. However, three patients showed NR to treatment and one developed infection. In addition, a combination of glucocorticoids and mycophenolate mofetil or mizoribine was administered to 7 patients. Immunosuppressants were contraindicated in 8 patients; hence, they received glucocorticoids alone, resulting in a remission rate of 80%. ARBs alone were administered to 17 patients in whom clinical symptoms were mild, and the remission rate was 66.7%. The 23 patients with MCD received glucocorticoids, among whom 11 and 6 patients achieved CR and PR, respectively, resulting in a remission rate of 89.5%. Two patients showed NR to treatment and one developed infection.

**Table 3.** Treatment and prognosis of common pathological types in the elderly between May 2015 and April 2017.

Types	Treat therapy	n	CR	PR	NR	lost to follow-up
*PGN*	immunosuppressive therapy	119	40	46	17	16
	ACEI/ARB	30	5	7	11	7
MN	Glucocorticoids + CTX	49	18	19	8	4
	Glucocorticoids + CSA/TAC	22	6	11	3	2
	Glucocorticoids + MMF/ MZR	7	2	3	0	2
	Glucocorticoids	8	0	4	1	3
	ACEI/ARB	17	2	6	4	5
MCD	Glucocorticoids	23	11	6	2	4
*SGN*	treat primary diseases	50	6	13	26	5
*TIN*	Glucocorticoids	7	4	2	1	0

CR: complete response; PR: partial response; NR: no response; PGN: primary glomerulonephritis; MN: membranous nephropathy; MCD: minimal change disease; SGN: secondary glomerulonephritis; TIN: tubular-interstitial nephropathy; CTX: cyclophosphamide; CSA: ciclosporin A; TAC: tacrolimus; MMF: Mycophenolate mofetil; MZR: mizoribine.

There were 50 cases of SGN, and they received specific treatment according to the primary disease; the remission rate was 42.2%. In addition, glucocorticoids were administered to the seven patients with TIN, and the remission rate was 85.7%.

## Discussion

In this study, we evaluated the clinical and pathological features of renal disease in 7,122 patients, with special emphasis on the 1199 elderly patients who underwent renal biopsy in a single center in Northeastern China. In addition to the clinical and pathological distribution analysis among different age groups, the incidence of complications of renal biopsy in both the elderly and non-elderly patients was noted. Furthermore, the treatment efficacy of different drugs in elderly patients was analyzed, of which reports are rare. Our center has performed the greatest number of renal biopsies in the Jilin Province of China; hence, our sample size was large enough for the purpose of this study. The findings of this study have important implications in advancing the understanding of the clinicopathological features of kidney disease in Northeast China and in deciding treatment protocol for these patients.

The percentage of elderly patients undergoing renal biopsy in our center is similar to that in previous reports [11]. As previously reported in multiple studies [12–16], the percentage of elderly patients undergoing renal biopsy, over the years, has increased in our center. Among elderly and non-elderly patients with renal disease, the risk of undergoing renal biopsy is comparable [6,17]. In addition, nephrologists may be more willing to perform biopsy in elderly patients in recent years compared to the past. In our study, the incidence of perirenal hematoma after biopsy was observed to be significantly lower in the elderly patients than that in the non-elderly patients. This may be explained by the less amount of activity 24 h after biopsy and better compliance. However, no significant difference was observed in relation to the incidence of complications (excluding perirenal hematoma) among elderly and non-elderly patients. No serious complications, including a requirement for blood transfusion or nephrectomy, developed. Hence, renal biopsy is a safe and valuable procedure for kidney disease diagnosis in elderly patients [18].

NS is the indication in approximately 40% of patients undergoing renal biopsy worldwide [19]. Most studies state NS as the most common clinical manifestation in elderly patients undergoing renal biopsy [6,20–22]. Zhu [20] and Uezono [23]  reported AKI as the most common manifestation following NS; however, another study reports CKD being more common than AKI [21]. In our study, in patients who underwent biopsy, the most common manifestation was NS, followed by GN, CKD, and AKI. Additionally, the incidence of GN was significantly lower in the elderly than that in the non-elderly patients; however, the incidences of NS, CKD, and AKI were significantly higher in elderly patients than those in non-elderly patients. It is also reported that patients above 80 years of age are more likely to develop AKI [24]. In our study, the incidence of AKI in elderly patients over 80 years was significantly higher those in the 60–69-year age groups. The pathological types of AKI were observed as SVARD, ATN, CreGN and MCD. The mean results of the laboratory investigations, in elderly patients, for blood albumin and proteinuria were 27.5 ± 9.1 g.L^−1^ and 5.2 ± 3.5 g.day^−1^, respectively, and are consistent to the findings of a previous study [25].

A meta-analysis of 176,355 patients from 15 provinces in China showed that 74% of patients in whom renal biopsy was performed had PGN [15]  . This was consistent with our study findings, as PGN was diagnosed in 70.8% of the elderly patients; however, the incidence of PGN was significantly lower in the elderly than that in the non-elderly patients. It is reported that the incidence rate of MN is on the rise [26] and MN is the most common pathological type of PGN in elderly patients in China [13,20,27,28], India [29], and Poland [30]. This was consistent with our study findings as the most common PGN was MN, followed by MCD and IgAN. In addition, the number of elderly patients with MN was significantly higher than that of non-elderly patients; this is assumed to be the result of the predilection of MN in the elderly [28,31]. IgAN was the common PGN [15], mainly in young adults. In our study, IgAN in elderly patients was significantly lower than in non-elderly patients, which is consistent with a previous study in Japan [22].

Previously, MPGN was thought to be prevalent in adolescents [32]. However, a recent study conducted in Japan found that the median age of the occurrence of primary MPGN was 59 years, and the peak age was 70 years [33]. This is consistent with our study findings, which showed the incidence of MPGN to be greater in elderly patients than in non-elderly patients. We believe that this may be related to the ethnicity and the age of the population.

The most common pathological type of SGN in elderly patients in our center was DN, which aligns with recent reports [28]. With the improvement of people's living conditions, the incidence of DN has significantly risen in elderly patients in recent years compared to previous findings [15]. Chen et al. [27] and Zhu et al. [20] reported SVARD as the most common SGN in elderly patients. In our study, the prevalence rate of SVARD was 4.8%, second only to DN (5.1%). The incidence of SVARD in elderly patients was significantly higher than that in non-elderly patients. Epidemiological studies have shown that the occurrence of vasculitis increases with age, peaking in the 65–74 year age group (60.1.million^−1^) [34].

Dysproteinemic kidney diseases are diseases in which B-cell or plasma cell clones produce pathogenic monoclonal immunoglobulins or light chains that cause kidney damage. This can manifest as amyloidosis, cast nephropathy, glomerular and tubular deposition diseases, and inflammatory glomerulonephritis [35]. In our study, there were 46 cases of dysproteinemic kidney diseases, mainly consisting of amyloidosis. In our study, the third most common SGN in elderly patients is amyloid nephropathy, the incidence rate of which is significantly higher in elderly patients than in non-elderly patients.

Elderly patients are prone to developing hypertension (HTN), and the kidney is one of the important target organs affected by HTN. Our data showed that the proportion of elderly patients with HTN was significantly higher than that of the non-elderly patients. Interestingly, MHTN was seen in fewer elderly patients than in non-elderly patients. A study in Nanjing, China, found that the average age of patients, in whom renal biopsy was performed, with malignant hypertensive renal damage was significantly lower than that of patients with benign hypertensive renal damage [36].

In our study, elderly patients were more likely to develop TIN (mainly acute and subacute) than non-elderly patients. The incidence rates of ATN and subacute TIN in elderly patients were higher than those in the non-elderly group. This is one of the reasons why the incidence of AKI in the elderly is higher than that in non-elderly patients. The elderly had significantly more drug-induced AIN (87% vs. 64%) than the younger patients [37].

The elderly patients were followed-up from May 2015 to April 2017. Consistent with the treatment of MN in adults [38,39], a combination of glucocorticoids and CTX was most commonly administered in elderly patients with MN. The 49 elderly patients who received this combination showed a remission rate of 82.2%. A combination of glucocorticoids and CSA or TAC was administered in 22 patients with MN, and a remission rate of 85% was observed. There was no significant difference in the remission rates between the two treatment methods in the elderly patients with MN, and a small number of the patients developed infection. Therefore, the treatment of MN in elderly patients should be decided on a patient-to-patient basis, and more attention ought to be paid to the prevention of infection. The second most common PGN in the elderly, MCD, was primarily treated by glucocorticoids in our study, and a high rate of remission (89.5%) was observed, with the development of infection in one case.

Renal biopsies can help in making a clear pathological diagnosis and in deciding the specific treatment regimen. Most of these diseases are difficult to cure, however, a 42.2% remission rate can be achieved. For instance, the elderly patients with a pathological diagnosis of TIN achieved 85.7% remission with steroid administration. Therefore, in the absence of contraindication, elderly patients with renal diseases ought to undergo renal biopsy as soon as possible, hence, they can receive optimal treatment with a greater chance of remission.

There are some limitations to our study. This is a retrospective study; hence, the number of patients followed up is limited. If more patients could have been followed up, more meaningful data might have been collected. Hence, further prospective studies are required to confirm the findings of this study. In addition, because the pathological manifestations of the patients are not single, there were some patients with coexisting pathological types, such as diabetic nephropathy with membranous nephropathy, in this study. However, they only accounted for 0.79% of the patients in this study. As excluding them from the analysis would affect the integrity of the study, we carefully analyzed the pathological and clinical manifestations of these cases and selected the most important pathological diagnosis for statistical analysis.

In conclusion, our study findings improve our understanding of the spectrum of kidney diseases among elderly and non-elderly patients in Northeast China and contribute significantly to the literature. From 2006 to 2020, the proportion of elderly patients who underwent renal biopsies increased every year. Renal biopsy is a relatively safe procedure in elderly patients. Furthermore, the findings of renal biopsy can help in deciding the choice of treatment and hence, can improve the prognosis.

## Data Availability

The datasets and resources generated and analyzed during the current study are available from the corresponding author upon reasonable request.
